# Inflammatory Mechanisms in the Pathophysiology of Diabetic Peripheral Neuropathy (DN)—New Aspects

**DOI:** 10.3390/ijms221910835

**Published:** 2021-10-07

**Authors:** Petra Baum, Klaus V. Toyka, Matthias Blüher, Joanna Kosacka, Marcin Nowicki

**Affiliations:** 1 Department of Neurology, University of Leipzig, Liebigstraße 20, D-04103 Leipzig, Germany; Petra.Baum@medizin.uni-leipzig.de; 2 Department of Neurology, University of Würzburg, Josef-Schneider-Str. 11, D-97080 Würzburg, Germany; kv.toyka@uni-wuerzburg.de; 3 Department of Medicine, University of Leipzig, Liebigstraße 20, D-04103 Leipzig, Germany; Matthias.Blueher@medizin.uni-leipzig.de; 4 Department of Visceral, Transplant, Thoracic and Vascular Surgery, University of Leipzig, D-04103 Leipzig, Germany; Joanna.Kosacka@medizin.uni-leipzig.de; 5 Institute of Anatomy, University of Leipzig, Liebigstraße 13, D-04103 Leipzig, Germany

**Keywords:** diabetic neuropathy, pathogenesis, inflammation, iron, treatment-induced neuropathy in diabetes (TIND)

## Abstract

The pathogenesis of diabetic neuropathy is complex, and various pathogenic pathways have been proposed. A better understanding of the pathophysiology is warranted for developing novel therapeutic strategies. Here, we summarize recent evidence from experiments using animal models of type 1 and type 2 diabetes showing that low-grade intraneural inflammation is a facet of diabetic neuropathy. Our experimental data suggest that these mild inflammatory processes are a likely common terminal pathway in diabetic neuropathy associated with the degeneration of intraepidermal nerve fibers. In contrast to earlier reports claiming toxic effects of high-iron content, we found the opposite, i.e., nutritional iron deficiency caused low-grade inflammation and fiber degeneration while in normal or high non-heme iron nutrition no or only extremely mild inflammatory signs were identified in nerve tissue. Obesity and dyslipidemia also appear to trigger mild inflammation of peripheral nerves, associated with neuropathy even in the absence of overt diabetes mellitus. Our finding may be the experimental analog of recent observations identifying systemic proinflammatory activity in human sensorimotor diabetic neuropathy. In a rat model of type 1 diabetes, a mild neuropathy with inflammatory components could be induced by insulin treatment causing an abrupt reduction in HbA1c. This is in line with observations in patients with severe diabetes developing a small fiber neuropathy upon treatment-induced rapid HbA1c reduction. If the inflammatory pathogenesis could be further substantiated by data from human tissues and intervention studies, anti-inflammatory compounds with different modes of action may become candidates for the treatment or prevention of diabetic neuropathy.

## 1. Introduction

The prevalence of diabetes and prediabetes has continued to increase worldwide in recent years [1,2,3,4,5]. Diabetes mellitus represents the most common cause of polyneuropathy with a prevalence of 18–49% in various clinical studies along with retinopathy, nephropathy, and vasculopathy [6]. Its prevalence seems to correlate with disease duration and glycemic control [7]. The most common and typical form of diabetic neuropathy (DN) is distal symmetric sensomotor and autonomic neuropathy, accounting for approximately 90% of patients [8]. In addition to the duration of diabetes, the prevalence also appears to be associated with other risk factors such as obesity and overt metabolic syndrome [9,10,11].

Small nerve fibers are considered to be most vulnerable structures for the development of DN since early damage was described even on patients with impaired glucose tolerance and without the expected overt chronic hyperglycemia [12].

Despite intensive research in recent years, there is still no unifying concept of the pathophysiology of DN nor efficacious and safe specific treatments available.

In this short review, we summarized the more recent aspects of two pathomechanisms based on our own experimental studies in diabetic disease models, namely inflammatory mechanisms and the modulation by manipulating systemic iron levels. These has been discussed against other recent findings and hypotheses. We conclude that re-examining these inflammatory mechanisms in human DN seems warranted.

## 2. The Neuropathogenic Role of Inflammation

In some rare forms of diabetic polyneuropathies, such as radiculoneuropathy in a nerve plexus distribution or cranial neuropathies, an inflammatory component has already been substantiated by nerve biopsies in distinct cases [8,13,14,15].

In our experimental studies, we could demonstrate that all animals with type 1 or type 2 experimental diabetes and neuropathy showed an increased inflammatory response, with mild infiltration and proliferation of macrophages and T cells as well as increased concentrations of soluble cytokines in the sciatic nerve [16,17,18]. Moreover, one of the predominant sites of axonal degeneration were the intraepidermal small nerve fibers [16,17,18]. This prompted us to propose that nerve tissue inflammation could be another facet in a multimodal pathogenic spectrum of causal factors in typical diabetic neuropathy.

Previously, increased inflammatory signs such as enhanced macrophage infiltration and proliferation in the peripheral nerves of rats with type 1 diabetes were shown [19]. In analogy, we found increased macrophage infiltration in the sciatic nerve of hyperglycemic *ob/ob* mice, a model of type 2 diabetes. This was associated with loss of myelinated and unmyelinated nerve fibers and axonal damage, indicative of overt neuropathy [20].

**Figure 1:**
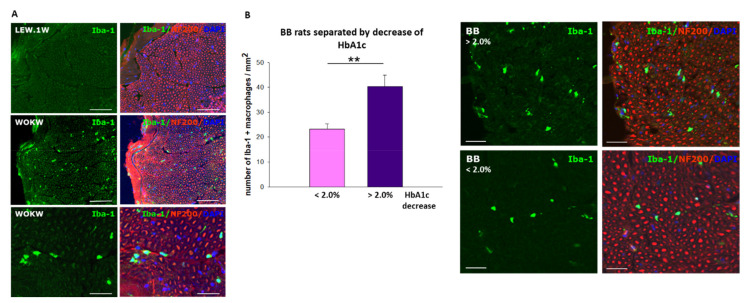
(**A**): Macrophages distribution in sciatic nerves of WOKW rats with metabolic syndrome and health LEW.1W control rats; [21]. Double immunofluorescence staining for Iba-1 (green, macrophages) and for neurofilament 200 (red). Nuclei are counterstained with DAPI (blue). Bar represents 100 µm and 50 µm (lower panel). (**B**): Macrophage distribution in sciatic nerves of BB/OKL rats stratified by the reduction in HbA1c values at the end of month 3 of insulin application [22]. Diagram: Quantification of Iba-1 (ionized calcium binding adaptor molecule 1)—positive macrophages. Photomicrograph: Double IF staining for Iba-1 (green, macrophages) and for neurofilament 200 (red). Note that the greater number of macrophages in sciatic nerve correlated with a larger reduction in HbA1c values (>2 per cent steps) as compared with the lesser reduction in HbA1c values (<2 per cent steps). Data from *n* = 7 are presented as mean ± SEM. ** *p* ≤ 0.01 according to the one-way analysis of variance together with the Newman–Keuls test. Bar represents 50 µm.

In a rat model of the metabolic syndrome (Wistar Ottawa Karlsburg W(RT1u) rats, WOKW), histopathological evidence of mild inflammation at the sciatic nerve was also found (Figure 1A) [21].

In a more recent study, we examined *ob/ob* mice without hyperglycemia as a model of the metabolic syndrome or prediabetes. Even in the absence of chronic hyperglycemia, symptoms of inflammation were evident in peripheral nerves [17].

In line with these experimental findings, it has been shown that type 2 diabetes and obesity or a combination of both may be associated with an increased inflammatory response [23]. Elevated serum concentrations of inflammatory biomarkers, e.g., C-reactive protein (CRP), interleukin (IL) 6, or IL 18 were found in humans with type 2 diabetes [24,25]. In addition, an association of biomarkers for inflammation with diminished cardiac autonomic function has been shown in type 2 diabetes [26,27].

Previously, in a prospective clinical study, in an elderly population with diabetes, Herder and co-workers [28] found that systemic biomarkers of inflammation were also linked to the onset and progression of neuropathy over 6.5 years of observation [28]. More recently, an increased expression of inflammation-associated genes has been observed in macrophages from dorsal root ganglia in DN patients [29]. The authors suggest that this mechanism may contribute to increased pain hypersensitivity. They also found downregulation in DRG-neuron-related genes. The elevated inflammatory gene profile and the accompanying downregulation of multiple DRG-neuronal genes provide new insights into intraganglionic pathology of DN in patients with type 2 diabetes [29].

The role of a mild nerve inflammation in primary non-inflammatory peripheral nerve disorders was first described in the pivotal experiments in transgenic mouse models of various inherited Charcot–Marie–Tooth (CMT) neuropathies almost 3 decades ago (reviewed in Martini and Toyka, 2004, [30]). These studies have shown that low-grade immune cell inflammation is an important pathogenic factor acting through macrophages and CD8 T cells. Formal proof of the pathogenic role of inflammatory pathways was provided in knockout mouse models lacking various activators of cell-based immune mediators [31,32,33]. In human hereditary neuropathies, low-grade inflammation was occasionally demonstrated in sural nerve biopsies, and a direct correlation with axonal damage was found [34,35].

In contrast, perivascular infiltration of macrophages and T cells in sural nerve and skin biopsies with increased proinflammatory cytokines are the culprit of nerve damage in a number of bona fide primary inflammatory neuropathies. These include vasculitic neuropathy, acute demyelinating inflammatory neuropathies (the Guillain-Barré syndrome, GBS), and chronic idiopathic demyelinating polyradiculoneuropathy (CIDP). Immune cell infiltration is more pronounced than in hereditary neuropathies and in diabetic neuropathy, and CD4-T cells are predominant in the immune-mediated neuropathies [30,36,37,38]. In the respective rat and mouse models of experimental autoimmune neuritis, it has been clearly shown that the cellular immune reactions were different from those in the transgenic CMT models [30,39].

**Figure 2:**
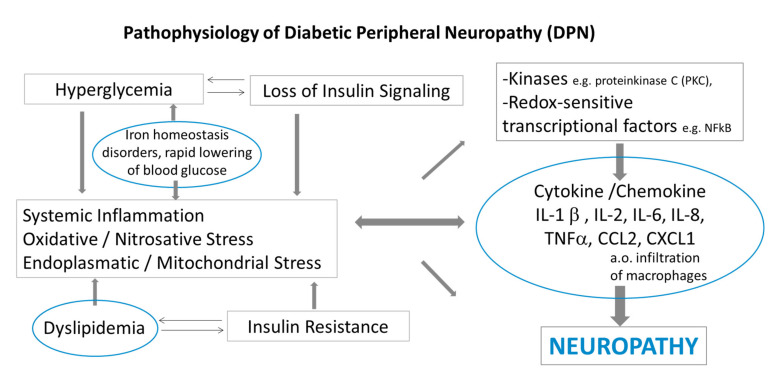
Pathophysiology of diabetic peripheral neuropathy, modified according to Pop-Busui, 2016 [40]. The relationship between inflammation in diabetes and damage of peripheral nerve fibers: hyperglycemia and insulin loss/resistance lead to dyslipidemia and oxidative/nitrosative stress of endoplasmic reticulum and mitochondria. These processes may contribute to accumulation of ROS (reactive oxygen species), inflammation and cellular damage. The infiltrated macrophages in the peripheral nerves trigger the production of pro-inflammatory factors: cytokine and chemokine, which promote inflammation and damage of nerve fibers. Disorders of iron homeostasis and rapid lowering of blood glucose enhances the inflammatory process of peripheral nerves and appears to affect hyperglycemia.

Based on these observations and that of Nukada et al. [19], we suggest that inflammation may indeed be an important factor in a multimodal pathway in the pathogenesis of diabetic neuropathy in addition to axonal degeneration of peripheral nerve fibers (Figure 2). It is worth considering these inflammatory pathways in terms of devising novel therapeutic approaches.

It should be emphasized here that peripheral nerve inflammation is associated with macrophage activation and this is leading to oxidative stress [41,42,43]. Increased concentrations of reactive oxygen species including mitochondrial overproduction of superoxide have also been linked to the development of diabetic microvascular complications, including experimental neuropathy [44]. Clinical studies in diabetic patients with or without neuropathy have shown an increase in the total oxidative status and in oxidative stress index levels together with a low total antioxidant status as compared with healthy individuals [45]. Moreover, other authors reported that the total antioxidant capacity in serum of patients suffering from DN was significantly decreased [46]. Strom et al. (2017) [47] demonstrated that patients with recently diagnosed type 1 and type 2 diabetes show evidence of systemic oxidative stress despite good glycemic control and they suggested a role of impaired extracellular antioxidative defense of superoxide in the early DN development [47]. Although peripheral neuropathy appears to be the most extensively investigated neuropathy in relation to oxidative stress, detailed studies of the role of oxidative stress in various animal models of diabetes are still limited.

## 3. Neuropathogenic Role of Iron Intake

A link between iron metabolism disorders and diabetes has already been proposed [48]. In several epidemiological studies, high serum ferritin and decreased transferrin levels were associated with a higher risk of type 2 diabetes, pre-diabetes, or gestational diabetes [49,50,51]. Iron overload may also lead to diabetes mellitus in hereditary hemochromatosis [52]. It has therefore been concluded that lowering of systemic iron levels by blood donation and iron chelators may be an option improving diabetes in patients [53,54]. This potential role of iron in the development of DN was not formally addressed by therapeutic intervention studies. 

Iron is an important co-factor for many vital functions such as oxygen transport, DNA synthesis and DNA repair, neurotransmitter synthesis, and myelination [55]. Iron deficiency is involved in the pathogenesis of peripheral neuropathies in children with iron deficiency anemia [56]. Iron excess, on the other hand, is thought to exert toxic effects and to have significance in the pathogenesis of neurodegenerative diseases such as Parkinson’s disease and Alzheimer’s disease [57,58]. While there are numerous studies on the importance of iron deposits in the central nervous system, to date, little is known about iron metabolism in the peripheral nervous system.

In an experimental study, we investigated the potential pathogenic role of exogenous non-heme iron in peripheral diabetic neuropathy (DN) in an animal model for type 1 diabetes [16]. Against our expectations, low rather than high-iron diet showed a strong effect on the development of experimental DN. Only a low iron-diet led to reduced sensory conduction velocities in the sciatic nerve. In addition, only STZ-rats on a low iron diet showed mitochondrial damage in numerous dorsal root ganglion (DRG) neurons [16].

**Figure 3:**
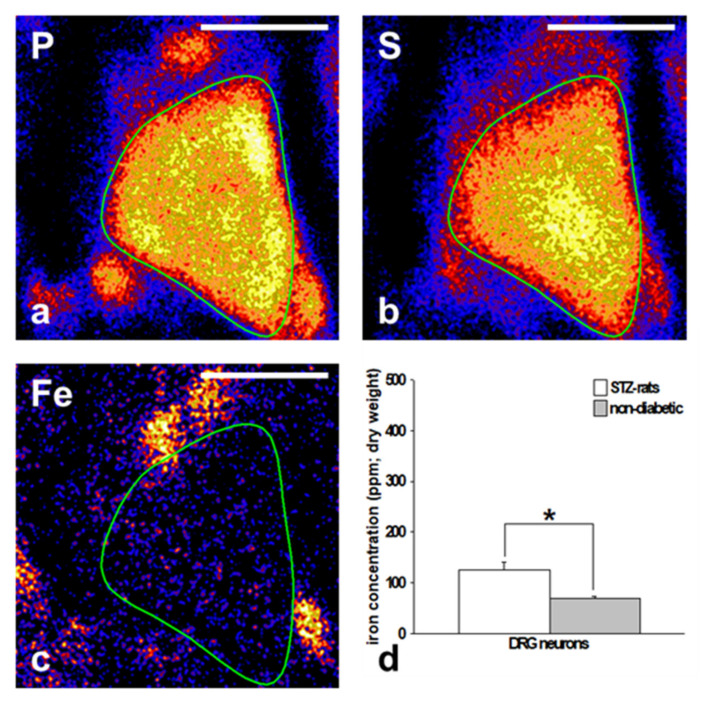

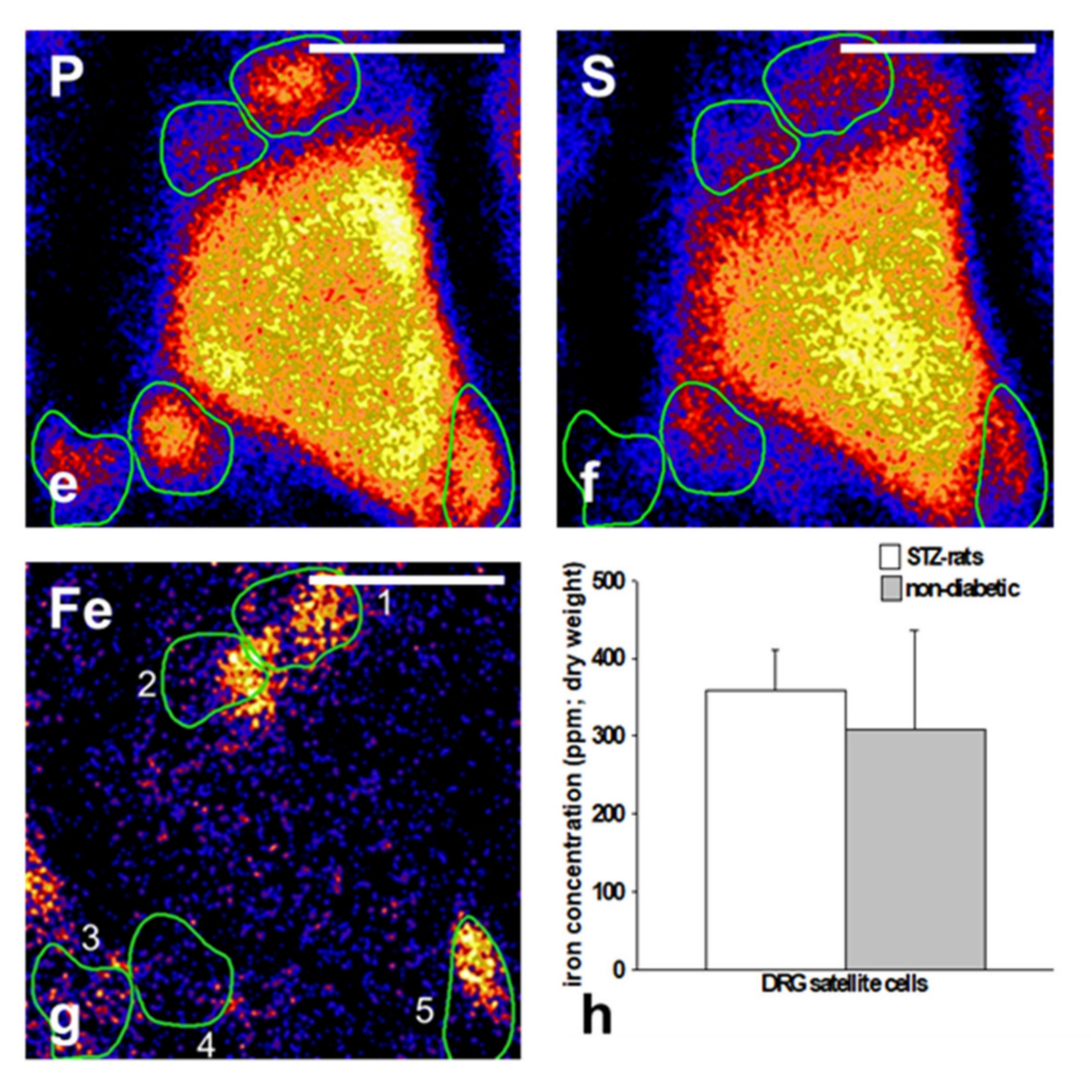
PIXE imaging of elementary phosphorous, sulfur (for cell area visualization) and iron particles in cells of dorsal root ganglions (DRG) [12]. (**a**–**c**): an example of the DRG neuron (marked with a drawn green line). (**e**–**g**): an example of DRG satellite cells (marked with a drawn green line). (**d**,**h**): Intracellular iron concentration: (**d**): The iron concentration was significantly higher in DRG neurons of standard diet STZ rats as compared with non-diabetic control rats. (**h**): There were no differences in the iron levels in satellite cells of STZ rats compared with non-diabetic control animals. Bars represent 20 µm.

Since diabetic rats developed sensory neuropathy on a low iron diet but did not show lowered systemic levels of iron, we concluded that DRGs and peripheral nerve fibers might be more vulnerable to dietary iron deprivation than other tissues (Figure 3).

Notably, we found comparable results in obese *ob/ob* mice with experimental metabolic syndrome [17] and in *db/db* mice with type 2 diabetes [18]. Similar to STZ-induced DN, low dietary iron load caused more pronounced abnormalities than high-iron load in *ob/ob* mice and *db/db* mice [17,18]. In contrast to the previous clinical and experimental observations indicating a negative effect of iron overload, we found that in high-iron fed diabetic mice, blood glucose and glycosylated hemoglobin A1c (HbA1c) concentrations were decreased rather than increased [18]. 

As a unifying finding, we demonstrated that all animals with type 1 or type 2 diabetes and those with metabolic syndrome showed an increased inflammatory response in the sciatic nerve in the iron-deficient group when compared with animals receiving a high-iron diet [16,17,18]. Notably, while numbers of pro-inflammatory M1 macrophages were reduced, anti-inflammatory M2 macrophages were increased in the sciatic nerve of *db/db* mice on high-iron diet as compared with low-iron diet animals [18].

In summary, dietary non-heme iron uptake seems to be a crucial factor in the pathogenesis of experimental diabetic neuropathy and also in the neuropathy associated with an experimental metabolic syndrome lacking overt diabetes. It remains to be explored to what degree the nerve pathology induced by low iron uptake is due to iron load itself or to the associated inflammatory activity in peripheral nerves.

## 4. Neuropathogenetic Role of Rapid Blood Glucose Lowering in Patients with Diabetes

A rare subtype of diabetic neuropathy was termed treatment-induced neuropathy in diabetes (TIND) because it was associated with a profound and often rapid iatrogenic reduction in hyperglycemia and HbA1c [59,60,61].

TIND is more common in individuals with longstanding hyperglycemia in type 1 diabetes. Although the primary clinical manifestation is neuropathic pain, there is a concurrent development of autonomic dysfunction both indicating small fiber neuropathy of thinly myelinated A-delta and of unmyelinated C-fibers. In addition, retinopathy and nephropathy are common complications [62].

In a retrospective study, clinical data from patients with diabetic neuropathy at a large diabetes center revealed that 11% suffered from TIND [63]. In these patients, TIND was accompanied by a marked decrease in HbA1c of more than 2 per cent steps over the previous 3 months [63].

The type of antidiabetic therapy (e.g., insulin, oral antidiabetic treatments or dietary interventions) did not seem to be relevant for the development of TIND. We are not aware of any prospective studies of the incidence of TIND in a defined cohort of patients nor of potential predictors of TIND. Since TIND involves degeneration of small nerve fibers standard electrophysiologic testing is not abnormal. Only by means of specific sensory tests (e.g., Quantitative sensory testing (QST) or nociceptive evoked potentials) and identifying epidermal nerve fiber damage by skin biopsy is helpful in identifying TIND-like pathology [35].

The pathogenesis of TIND is still incompletely understood [64,65]. Low and Singer [64] proposed a concept of “energy crisis” brought about by chronic endoneural edema. In a diabetes animal model, they showed that chronic hyperclycemia leads to an increased intercapillary distance, resulting in reduced nerve blood flow and subsequently in a long-standing hypoxic endoneural microenvironment. Due to the proposed poor blood flow autoregulation in peripheral nerves the nerve fibers could be susceptible to changes in the metabolic milieu [64,66].

Recently, we addressed the potential mechanisms in TIND by developing an animal model in BB rats (bio breeding/ OKL, Ottawa Karlsburg Leipzig, BB/OKL). BB/OKL rats are an established animal model for type 1 diabetes. We induced a rapid and profound decrease in HbA1c by insulin treatment aiming at mimicking the situation in TIND [22]. We found significantly higher abnormalities in motor and compound sensory nerve conduction velocities and a significantly greater infiltration of macrophages in sciatic nerves (Figure 1B). This was associated with a reduced number of calcitonin gene-related peptide (CGRP)-positive nerve fibers as compared with the animals with a milder decrease in HbA1c. We concluded that a mild acute neuropathy with inflammatory components was induced in BB/OKL rats as a consequence of the abrupt decrease in HbA1c caused by high-dose insulin treatment. In contrast to TIND in patients, there was an obvious nerve conduction slowing indicating that large, myelinated nerve fibers were also afflicted, in addition to small fibers [22].

Supportive care is currently the only available treatment of TIND. More research is required to further elucidate the various pathogenic pathways in TIND ultimately leading to better prevention and treatment of this rare complication.

## 5. Neuropathogenic Role of Dyslipidemia

Type 2 diabetes is usually associated with dyslipoproteinemia, which may also play a role in the development of diabetic neuropathy. Obesity alone may increase the risk of developing polyneuropathy [67].

In a clinical study in obese children and adolescents, we demonstrated extended autonomic nervous system (ANS) dysfunction, affecting several organs with a dysfunctional activity of parasympathetic and sympathetic nervous systems. The pattern of autonomic dysfunction raises the possibility that obesity may give rise to autonomic dysfunction of peripheral nerves resembling that observed in normal-weight diabetic children and adolescents [68]. To date, there are no studies on nerve biopsies or skin biopsies that might further elucidate nerve pathology.

Pathogenic mechanisms similar to those in the pathogenesis of diabetic neuropathy may be candidates for further studies [69].

Leptin-deficient *ob/ob* mice have been described as a model for obesity and the metabolic syndrome that may be associated or not with mild diabetes [70].

In our first study using this animal model, we found pathological changes in nerve fibers and their endoneural microvessels, especially in distal parts of the sciatic nerves. Notably, this pathology was present even if there was no overt diabetes [71].

Experimental data from a pilot study obtained in high-fat diet fed mice with profound obesity showed a marked and statistically significant drop in compound muscle action potential amplitudes but no conduction slowing indicating functional abnormalities typical of an axonal neuropathy (Grünewald B, Toyka KV, Höke A, 2013, unpublished observations).

In our recent study in *ob/ob* mice, we detected mild to moderate inflammatory activity in peripheral nerves [17]. We suggest that DN may be triggered or even induced by obesity or dyslipidemia.

**Figure 4:**
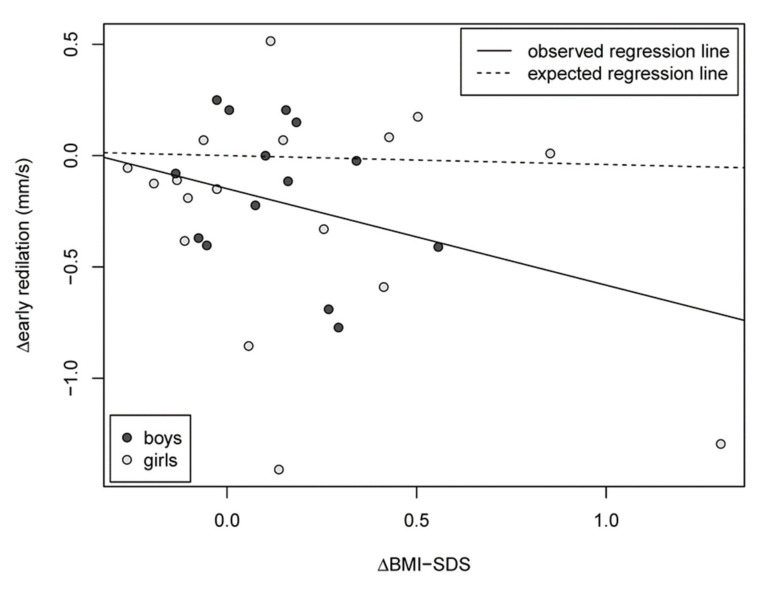
Associations between changes in BMI-SDS and changes in early re-dilatation velocity in in quantitative pupillography taken as a parameter for autonomic sympathetic dysfunction. BMI—body mass index; SDS—standard deviation score [72].

In one of our clinical studies, we found stabilization and improvement of autonomic neuropathy in correlation with improvement of weight status and metabolic risk factors after lifestyle-intervention, i.e., a combination of regular exercise (150 min/week, nutritional counseling, and psychological support (Figure 4) [72].

We concluded that autonomic nervous dysfunction may be positively influenced by an intervention leading to weight loss, even as early as during childhood, an observation that may translate to improved cardiovascular health and mortality later in life [72].

Another important aspect is that physical activity in humans including aerobic, resistance training and especially combined exercise may improve inflammation-linked biomarkers and thus suggests an anti-inflammatory effect through these interventions [73,74].

## 6. Conclusions

The findings from experiments using animal models of type 1 and type 2 diabetes and using animal models for obesity and the metabolic syndrome lead us to suggest that low-grade intraneural inflammation may be a major facet of the common types of diabetic neuropathy. If the inflammatory phenomena observed in animal experiments could be confirmed as a pathogenic principle of DN in humans, anti-inflammatory therapies would become a candidate for anti-inflammatory therapeutic strategies. Based on the profound intraepidermal small fiber degeneration as shown in our animal models, minimally invasive skin biopsies are recommended in future prospective trials aimed at defining inflammatory mechanisms.

Neurodegeneration along with inflammatory activity caused by iron deficiency has not yet been investigated systematically in humans. In practical terms, our experimental observations lead us to discourage the use of iron-reducing therapies in diabetes until more is known about the effects of iron intake in patients and until these human studies have been done.

In a new animal model mimicking TIND in diabetes, the affliction of small nerve fibers could be reproduced and associated with inflammatory pathology, and this may be a consequence of an abrupt lowering of HbA1c. Still, the exact pathomechanism needs to be defined in future studies before novel treatment strategies can be proposed.

## Data Availability

Not applicable.
